# Preoperative vs Postoperative Patient Outcome and Recovery Expectations of Total Joint Arthroplasty

**DOI:** 10.1016/j.artd.2025.101626

**Published:** 2025-02-11

**Authors:** Siddhartha Dandamudi, Kyleen Jan, Madelyn Malvitz, Anne DeBenedetti, Omar Behery, Brett R. Levine

**Affiliations:** a Department of Orthopaedics, Rush University Medical Center, Chicago, IL, USA; b MedStar Georgetown University Hospital, Georgetown University School of Medicine, Washington, DC, USA

**Keywords:** Total joint arthroplasty, Satisfaction, Expectations, Mobility and pain

## Abstract

**Background:**

Patient satisfaction with total joint arthroplasty (TJA) remains a challenge, with up to 20% of patients expressing dissatisfaction despite good clinical outcomes. This study aims to assess patient expectations and experiences prior to and after undergoing a primary TJA.

**Methods:**

A 13-question survey assessing patient expectations around surgical risks, logistics, physical therapy (PT), and pain was distributed in the clinics of multiple surgeons at an academic center. Each patient was categorized as either preoperative or postoperative, with similar questions for both groups. No identifying information was collected.

**Results:**

One hundred eight preoperative and 344 postoperative responses were collected. Preoperatively, 91.3% of patients felt they had enough time to ask questions compared to 98.5% (*P* = .01) postoperatively. Preoperative patients named risks of TJA more accurately (*P* < .00001). Expectations of long-term pain differed: 48.3% of preoperative patients expected no pain and 1.7% expected to be unable to move; postoperatively, 7.3% (*P* < .0001) reported no pain and 10.3% (*P* = .03) were unable to move; 17.3% of patients finished PT within 2 weeks, compared to 1.7% who believed this was possible (*P* =.0027). A total of 73.1% of postoperative patients requested additional weeks of PT. Only 52.1% or patients had a long-term follow-up plan in place.

**Conclusions:**

Expectations vary in patients undergoing TJA. Preoperative patients may have unrealistic expectations regarding postoperative pain and mobility. The need for more PT and the lack of a long-term follow-up plan highlight the importance of comprehensive perioperative communication to align expectations and potentially improve satisfaction and follow-up compliance.

## Introduction

The American Joint Replacement Registry estimated more than 2.8 million total hip and knee arthroplasties were performed in the United States in 2022 [1]. Based on data from the US National Inpatient Sample combined with Census Bureau, this number is expected to rise for total hip and knee surgeries by 284% and 401%, respectively [2]. This growth in volume may be attributed to public-facing opinions of the positive impact of total joint replacement on patient quality of life, with both hip and knee arthroplasty demonstrating cost-effective and impactful clinically significant improvements in patient joint pain and clinical function outcomes [3,4]. Nonetheless, patient satisfaction with total joint arthroplasty (TJA) remains multifactorial, with notable proportions of unsatisfied patients, particularly with total knee arthroplasty (TKA) dissatisfaction proportion of 12.7% 5 years post-TKA in 1 study [5]. Other studies indicated that this percentage dissatisfied patients may be as high as 20% [[6], [7], [8]].

A meta-analysis of 19 studies reported satisfaction rates were approximately 75% at 5 years postoperatively for total knee replacements [9]. Dissatisfaction was correlated to an insufficient quality of life, including activity and social limitations, as well as physiological and emotional aspects [10]. With approximately 25% of patients reportedly not achieving satisfaction, it is important to understand the contributing factors providing this level of dissatisfaction. Such factors likely include mismatches for patient expectations of postoperative pain, difficulty of the recovery, and final functional levels. The study suggested dissatisfaction may be related to patient-related factors, including age, gender, obesity, medical comorbidities, ethnicity, and psychological status [10]. However, other patient-centric factors, specifically, patient-provider communication and patient expectations surrounding surgery, are not adequately studied but anecdotally play a critical role in patient satisfaction following TJA. Furthermore, there is negligible information surrounding the limitations in patient-provider communication on various perioperative aspects with TJA, including physical therapy (PT).

With the increasing volume of TJAs and growing emphasis on patient-reported clinical outcomes, a better understanding of the relevant patient perspectives and expectations surrounding surgery may prove useful in guiding surgeons to improve patient clinical outcomes and satisfaction postoperatively. This study aims to ascertain patient perspectives and expectations before and after undergoing TJA to identify potential targets for patient dissatisfaction around arthroplasty which clinicians can consider in the delivery of care.

## Material and methods

Following approval from the institutional review board, a single-center cross-sectional descriptive study was created. A 10-question survey aimed to assess preoperative expectations, and a 13-question survey aimed to assess postoperative experiences were developed by the senior authors. Inclusion criteria for the preoperative survey were all new and established patients who have not had TJA and for postoperative survey were all patients who have had TJA more than 6 weeks prior to survey administration. All patients were given the choice to opt out of participating in the survey. Any patient who chooses not to fill the survey out was excluded. Over a 3-month period, surveys were distributed to patients of 5 fellowship-trained arthroplasty surgeons at a single, high-volume, tertiary care academic center in the clinical setting. Prior to their regularly scheduled appointment, patients received appropriate surveys from the front desk staff. Instructions were given to the patients to not write any identifying information on other surveys and the front-desk staff collected the paperwork prior to rooming of the patient. No correlation of specific responses to specific patients was done by authors.

Surveys were written and distributed with the goal to compare expectations of preoperative patients planning to undergo TJA with postoperative patient experiences on several aspects of perioperative care. The first batch of questions were aimed at assessing if patients felt enough time was afforded to ask questions about the surgery. The second part of the surveys looked to elicit if patients were understanding the risks of surgery, their expected hospital stay, and expected recovery time. The final part of the survey looked to assess PT experiences, pain, and functional level expectations of respondents.

All surveys were collected from a collection box and then extracted to Microsoft Excel (Version 16.85, Microsoft, Redmond, WA) for descriptive data analysis. Qualitative data are presented as percentages of respondents and a 2-proportion test was used to assess differences between the 2 cohorts. A *P* value of less than .05 was considered significant.

## Results

Responses were collected from 108 patients preoperatively and 344 patients postoperatively. Questions asked of respondents are seen below in Table 1. Table 2 demonstrates the breakdown of surgery type and location of the operation.

**Table 1:** Questions compared before and after surgery.

Questions
1. What surgery are you having/*did you have*?
2. Where are you having/*where did you have surgery*?
3. How long were you willing to wait to have surgery?^a^
4. Prior to surgery, did you have adequate time to ask your surgeon/*surgeon team questions*?
5. Which of the following are risks of total hip/knee replacement?
6. How long do you expect to stay/*did you stay in the hospital/surgical center after your surgery*?
7. How long do you anticipate it taking to recover after surgery/*did it take to recover*?
8. How long do you think physical therapy will last/*how many weeks did you complete*?
9. How many more weeks of physical therapy do you think you need?^b^
10. After fully recovering, what do you anticipate your long-term pain level to be/*what was your pain level after surgery*?
11. Based on your preoperative activity status, what are your expectations for/*how active are you after surgery*?
12. Did you have adequate opportunity to talk to your surgeon since your surgical procedure?^b^
13. Which visit is the most important to see your surgeon?^b^
14. Did your surgeon offer a long-term follow-up plan?^b^

All italicized questions were asked on postoperative survey.

a Question only asked on preoperative survey.

b Questions asked only on postoperative survey.

**Table 2:** Surgery type and location of operation from respondents.

Parameter	Presurgery %	Postsurgery %	*P* value
Surgery type			
Total hip arthroplasty	27.78	35.76	.12
Total knee arthroplasty	58.34	55.23	.57
Both	2.78	9.01	**.03**
None	11.11	-	-
Location			
Hospital	47.57	81.29	**<.001**
Hospital-based surgery center	10.68	7.02	.23
Free-standing surgery center	8.74	11.43	.45
Not sure	33.01	0.29	**<.001**

Bold values indicate statistical significant (*P* < .05).

A significant portion of postoperative patients had both total hip arthroplasty and TKA (*P* = .03), as shown in Table 2. Many patients (33.0%) preoperatively did not know what type of institution they were going to have their surgery performed (*P* < .001). Additionally, a significant difference was seen in the proportion of patients who knew they were having surgery at a hospital preoperatively and the percentage of patients who actually did have a hospital-based procedure (*P* < .001). Preoperative patients reported a 90.4% rate of having adequate time for questions, which was significantly lower than the 98.5% reported by postoperative patients (*P* = .01).

Table 3 illustrates the breakdown of risk factor awareness before and after surgery. Overall, there was a difference between preoperative (40.7%) and postoperative (34.4%) patients in acknowledging the risks of TJA (*P* < .0001). Significant differences were seen with more preoperative patients naming extended hospitalization (*P* < .001) and pneumonia (*P* = .02) as potential risks compared to postoperative respondents. Other specific risks including infection, instability, loss of limb, death, implant loosening, continue pain, and blood clots demonstrated a higher percentage of preoperative patients naming these risks, although none are significant (*P* > .07). Postoperative patients did identify the risk of blood transfusions (31.4%) compared to preoperative (26.6%) patients, but this was not statistically significant (*P* = .34).

**Table 3:** Percentage of patients stating they were aware of (presurgery) and had discussed (postsurgery) certain risks of surgery.

Risk	Presurgery %	Postsurgery %	*P* value
Infection	82.57	81.27	.76
Instability (dislocation)	33.94	29.68	.40
Fracture of the bones during or after surgery	33.94	27.09	.17
Loss of limb	22.02	14.70	.07
Death	33.03	21.33	.12
Implant loosening	34.87	26.22	.08
Continued pain	50.46	44.09	.24
Pneumonia	23.85	14.70	**.02**
Blood clots	65.14	64.84	.95
Extended hospitalization	41.28	22.47	**<.001**
Blood transfusion	26.61	31.41	.34

Bold values indicate statistical significant (*P* < .05).

Table 4 outlines the expected and actual experiences of preoperative and postoperative patients in hospital stay time following TJA, recovery time, PT, pain level following surgery, and expected postoperative activity level. Differences in expected stay in the hospital after TJA were seen with more preoperative patients (30.1% vs 19.9%) believing their stay would be less than 8 hours (*P* = .05) and more postoperative respondents (26.7% vs 14.6%) staying 2 days (*P* < .001). Regarding anticipated recovery, an overall trend was seen that 87.4% of preoperative patients believed their recovery would be approximately 3-6 while 74.7% of postoperative patients indicated this timeline fit their recovery pattern. A significantly higher proportion (*P* = .04) of postoperative patients (7.6%) stated their recovery took 1 year, while only 1.9% of preoperative patients indicated that they were expecting recovery to take at least 1 year. Differences in PT support postoperatively were also noted. Preoperative patients anticipated a significantly shorter amount of PT, with 31.7% expecting 4-6 weeks and 34.6% anticipating 6-8 weeks, compared to postoperative patients, where only 16.3% participated in 4-6 weeks of PT (*P* = .006) and 24.1% underwent 6-8 weeks (*P* = .04) of PT. Postoperative patients reported significantly longer stints of PT, with 37.62% requiring 2 months or more, compared to preoperative expectations, where only 21.2% anticipated needing 2 or more months (*P* = .02).

**Table 4:** Time expected to stay in the hospital, time to recover from surgery, time to attend physical therapy, level of pain, and expectations of activity level obtained presurgery versus actual time spent obtained postsurgery.

Time	Presurgery %	Postsurgery %	*P* value
Stay in hospital			
< 8 h	30.10	19.89	**.05**
8-12 h	11.65	7.41	.18
1 d	29.12	28.79	.94
2 d	14.56	26.71	**<.001**
1 wk	6.80	12.17	.13
> 1 wk	4.85	5.04	.94
Recovery			
4-6 wk	24.27	17.38	.12
6-12 wk	33.98	25	.07
3-6 mo	29.18	32.32	.54
6-12 mo	10.68	17.68	.09
>1 y	1.94	7.62	**.04**
Physical therapy			
<2 wk	0.96	5.01	.07
2-4 wk	11.54	16.93	.19
4-6 wk	31.73	16.30	**.006**
6-8 wk	34.62	24.14	**.04**
>2 mo	21.15	37.62	**.02**
Pain level			
No pain	51.92	8.45	**<.001**
Mild occasional pain	19.23	38.67	**.0002**
Intermittent pain with certain activities	27.88	40.18	**.02**
Unable to move because of pain	0.96	12.69	**.0004**
Expectations of activity level			
Should be able to resume all prior activities	52.88	40.42	**.03**
Anticipate some mild limitations	31.73	44.07	**.03**
Anticipate a significant change in what I can do	15.38	15.50	.97

Bold values indicate statistical significant (*P* < .05).

Pain-level expectations varied greatly between the 2 cohorts. Reporting of no pain after TJA saw a significant difference between the groups (*P* < .001) with 51.4% of preoperative patients expecting no pain, while only 8.5% of postoperative patients reported this outcome. Reports of mild occasional pain (*P* = .02), intermittent pain with certain activities (*P* = .02), and unable to move because of pain (*P* = .0004) all demonstrated significantly higher rates in the postoperative group compared to the preoperative group. Less than 1 of patients thought they would be unable to move due to pain (0.96%), yet postsurgery 12.69% of patients reported this outcome. Activity-level trends were similar to pain-level trends. The majority of preoperative patients (52.9%) believed they should be able to resume all prior activities after TJA, with a significantly lower (*P* = .03) rate of 40.4% reported from postoperative patients. A greater proportion of postoperative patients reported mild activity limitations, with 44.1% compared to 31.2% in the preoperative group (*P* = .03). No differences were seen in the rate of patients anticipating significant changes in their activity level.

Preoperatively, 63% of patients were only willing to wait up to 10 weeks to undergo the necessary joint replacement. Of respondents, 91.3% of preoperative patients indicated that they had adequate time to ask questions, while 97.4% (*P* = .009) stated they did have adequate time. Furthermore, 99.3% of patients indicated they had enough face time with the surgeon postoperatively. Regarding PT, 53.2% of postoperative respondents stated that they would like 4 to more than 6 more weeks of therapy on top of the base therapy prescribed. Finally, only 53.4% of postoperative respondents indicated long-term follow-up was planned.

## Discussion

Through assessing expectations and experiences of patients who are undergoing and have gone through TJA, our study aimed to identify gaps in patient satisfaction and areas of improvement in clinical care within hip and knee arthroplasty. Our findings reveal several areas of conflict between patient expectations and postoperative experience, particularly regarding assessment of risks of TJA, expectations around pain and activity level, and PT. These conflicts highlight the need for further research into perioperative patient expectations, as well as the most effective methods and strategies for counseling patients to ensure alignment between expectations and outcomes.

Patient awareness of risk factors seemed to be lacking both preoperatively and postoperatively. Risk factors such as instability (dislocation), fracture during or after surgery, loss of limb, death, implant loosening, continued pain, pneumonia, extended hospitalization, and blood transfusion all had awareness less than 50% both before and after the surgery (Table 3). This low level of awareness could stem from insufficient communication by surgical teams or the possibility that patients, particularly of older age, may struggle to process complex medical information during stressful times [11]. Kearney et al [12] discussed the impact of preoperative preparation classes indicating these interventions impacted patient experience and preparedness heading into surgery. Improving patient education on risk factors both before and after surgery could enhance their ability to make informed decisions about their care, thereby potentially increasing satisfaction.

Postoperative pain is another factor that is understood to be a consequence of surgery, but the level does not correlate with its expectation. A study by Blom et al [13] showed that 7%-23% of patients experience moderate or severe pain following their TJA. Before surgery, a substantial 51.9% anticipated having no pain after TJA, yet only 8.45% reported this outcome after surgery. On the other end of the pain spectrum, 0.96% expected to be unable to move because of pain preoperatively, and 12.69% reported this after surgery. This discrepancy is highlighted through expectations of activity level as well with 31.7% of patients expected to be able to resume all prior activities without limitation; only 40.4% of patients postoperatively felt they could return to all previous activities. Arpey et al [14] illustrated that patient expectations are often 2 times higher than functional measurements of success in TJA. These mismatches of functional and patient beliefs likely contribute to the dissatisfaction rate of 20% for TJA [6,14].

PT after a hip replacement has been shown to improve physical function at 3-4 months and decrease pain compared to those who do not participate [15,16]. While several studies have demonstrated that formal therapy is not always necessary after TJA, our study found that most patients underestimated the time required for PT and more than half of patients (53.2%) requested 4-6 more weeks of PT than initially prescribed [[17], [18], [19]]. Only 18.3% of patients expected their PT to last longer than 2 months, yet 44.4% had already completed more than 6 weeks of therapy when surveyed postoperatively, with 26.1% expecting to need an additional 6 weeks. Jones et al [20] demonstrated that preoperative PT classes have significantly improved functional outcomes, decreased length of stay, and savings of around $900 in costs related to the TJA. The importance of PT cannot be understated, and clinicians should consider emphasizing it preoperatively, but also using shared decision-making to optimize the amount of PT postoperatively.

Follow-up appointments postsurgery are extremely important to identify complications early in their progress. Compliance to attend these appointments is often low from patients, with a study by Clohisy et al [21] showing only 61% of patients present to the office for their 1-year follow-up and 36% at their 2-year follow-up. Due to this, it is pertinent that offices dedicate effort toward scheduling these appointments and sending adequate reminders to emphasize their importance. In this study, only 52.1% stated long-term follow-up was planned, showing more work is needed to make this a priority in their care. There is room for improvement in how follow-up care is communicated and prioritized. Efforts to schedule these appointments and provide adequate reminders could help to emphasize their importance and improve patient outcomes.

While this study provides valuable insights, there are limitations to acknowledge. The presurgery and postsurgery surveys were not matched per patient, meaning we could not directly compare individual expectations and outcomes. Additionally, this study is a cross-sectional survey design which was conducted at a single, academic center, which may limit the generalizability of these findings and carries the limitations of convenience sampling bias.

## Conclusions

Patients undergoing TJA have a range of opinions and expectations on what their outcome will be. Often, these expectations stated preoperative realities do not align with postoperative realities. This disconnect is evident in areas such as pain expectations postoperatively, PT timelines, and awareness of surgical risks. Even more, the long-term follow-up planning in arthroplasty clinics is not clear to patients. These findings shed light on the need for more comprehensive perioperative communication between patient and provider. Changes to improve alignment to postoperative expectations preoperatively could increase overall patient experience and satisfaction.

## Conflicts of interest

Brett Levine received IP royalties from Link Orthopedics; is a paid consultant for Enovis, Link Orthopedics, Merete, and Zimmer Biomet; received research support from Zimmer Biomet as a Principal Investigator; is in the editorial or governing board of Arthroplasty Today, Elsevier, Human kinetics, SLACK Incorporated, and Wolters Kluwer Health - Lippincott Williams & Wilkins; and is a board or committee member of AAOS, American Association of Hip and Knee Surgeons, Knee Society, and MAOA. Anne DeBenedetti is a paid consultant for JointMedica Limited. All other authors declare no potential conflicts of interest.

For full disclosure statements refer to https://doi.org/10.1016/j.artd.2025.101626.

## CRediT authorship contribution statement

**Siddhartha Dandamudi:** Writing – review & editing, Writing – original draft, Visualization, Validation, Supervision, Project administration, Methodology, Investigation, Formal analysis, Data curation, Conceptualization. **Kyleen Jan:** Writing – review & editing, Writing – original draft, Visualization, Methodology, Investigation, Formal analysis, Data curation, Conceptualization. **Madelyn Malvitz:** Writing – review & editing, Writing – original draft, Project administration, Methodology, Investigation, Formal analysis, Data curation, Conceptualization. **Anne DeBenedetti:** Writing – review & editing, Writing – original draft, Supervision, Project administration, Data curation, Conceptualization. **Omar Behery:** Writing – review & editing, Writing – original draft, Supervision, Project administration, Methodology, Investigation, Conceptualization. **Brett R. Levine:** Writing – review & editing, Writing – original draft, Visualization, Validation, Supervision, Project administration, Methodology, Investigation, Formal analysis, Data curation, Conceptualization.

## Appendix ASupplementary data

Conflict of Interest Statement for DeBenedetti

Conflict of Interest Statement for Malvitz

Conflict of Interest Statement for Behery

Conflict of Interest Statement for Levine

Conflict of Interest Statement for Jan

Conflict of Interest Statement for Dandamudi
