# Supplementary material for: Preoperative vs Postoperative Patient Outcome and Recovery Expectations of Total Joint Arthroplasty
Source: Arthroplast Today. 2025 Feb 11;32:101626. doi: 10.1016/j.artd.2025.101626 (PMC11869528; doi:10.1016/j.artd.2025.101626)
Supplement: Conflict of Interest Statement for Levine [file mmc4.pdf]

CONFLICT OF INTEREST STATEMENT

American Association of Hip and Knee Surgeons

(Adopted from the American Academy of Orthopaedic Surgeons disclosure statement)

Preoperative versus Postoperative Patient Outcome and Recovery Expectations of Total Joint Arthroplasty

Manuscript Title

1. Royalties from a company or supplier (The following conflicts were disclosed)

   Link Orthopedics: IP royalties

2. Speakers bureau/paid presentations for a company or supplier (The following conflicts were disclosed)

   None

3A. Paid employee for a company or supplier (The following conflicts were disclosed)

   None

3B. Paid consultant for a company or supplier (The following conflicts were disclosed)

   Enovis: Paid Consultant
   Link Orthopedics: Paid Consultant
   Merete: Paid Consultant
   Zimmer Biomet: Paid Consultant

3C. Unpaid consultants for a company or supplier (The following conflicts were disclosed)

   None

4. Stock or stock options in a company or supplier (The following conflicts were disclosed)

   None

5. Research support from a company or supplier as a Principal Investigator (The following conflicts were disclosed)

   Zimmer Biomet: Research Support

6. Other financial or material support from a company or supplier (The following conflicts were disclosed)

   None

7. Royalties, financial or material support from publishers (The following conflicts were disclosed)
   None

8. Medical/Orthopaedic publications editorial/governing board (The following conflicts were disclosed)

   Arthroplasty Today: Editorial or Governing Board
   Elsevier: Editorial or governing board
   Human kinetics: Editorial or governing board
   SLACK Incorporated: Editorial or governing board
   Wolters Kluwer Health - Lippincott Williams & Wilkins: Editorial or governing board

9. Board member/committee appointments for a society (The following conflicts were disclosed)

   AAOS: Board or Committee Member
   American Association of Hip and Knee Surgeons: Board or Committee Member
   Knee Society: Board or Committee Member
   MAOA: Board or Committee Member

<u>Each author must sign AND print or type his/her name, date and submit a separate form</u>

In addition, one BLINDED Conflict of Interest form (no author names used) should be submitted per manuscript with all author disclosures.

Brett Levine		09/18/2024
Author Name (Print or Type)	Author Signature	Date
